# Human Face Perception Using Electroencephalography and Magnetoencephalography

**DOI:** 10.3389/fphys.2022.803274

**Published:** 2022-03-31

**Authors:** Kensaku Miki, Yasuyuki Takeshima, Shoko Watanabe, Ryusuke Kakigi

**Affiliations:** ^1^Integrative Physiology, College of Nursing, Aichi Medical University, Nagakute, Japan; ^2^School of Nursing, Japanese Red Cross Toyota College of Nursing, Toyota, Japan; ^3^Department of Integrative Physiology, National Institute for Physiological Sciences, National Institutes of Natural Sciences, Okazaki, Japan; ^4^Higashi Owari National Hospital, National Hospital Organization, Nagoya, Japan

**Keywords:** electroencephalography (EEG), magnetoencephalography (MEG), face, N170, inferior temporal (IT), superior temporal sulcus (STS)

## Abstract

The face has a large amount of information that is useful for humans in social communication. Recently, non-invasive methods have been used to investigate human brain activity related to perception and cognition processes. Electroencephalography (EEG) and magnetoencephalography (MEG) have excellent temporal resolution and reasonably good spatial resolution. Therefore, they are useful to investigate time sequences of human brain activity related to the face perception process. In this review, we introduce our previous EEG and MEG studies of human face perception that demonstrated the following characteristics of face perception processing: (1) Event-related components in the temporal area related to the activity in the inferior temporal (IT) area, corresponding to the fusiform face area (FFA), are evoked approximately 180 msec after the presentation of a face. The activity in the IT area plays an important role in the detection processing of a face, and the contours of a face affect the activity in the IT areas. (2) Event-related components in the temporal area related to the superior temporal sulcus (STS) activity are larger when eyes are averted than when directly looking into the eyes. (3) The direction of features of a face affects the face perception processing in the right hemisphere. On the other hand, the matching of the direction between the contours and features of a face affects the processing in the left hemisphere. (4) Random dots blinking (RDB), which uses temporal changes in patterns of many small dots to present stimuli without a change in luminance during the presentation of a face, is a useful visual stimulus method to investigate the brain activity related to face perception processing in the IT area using EEG and MEG.

## Highlights

In this review, we discuss the following characteristics of face perception processing based on our previous electroencephalography (EEG) and magnetoencephalography (MEG) studies:

– Event-related components related to the activity in the inferior temporal (IT) area are evoked after the presentation of a face. In addition, the activity in the IT area plays an important role in the detection processing of a face and the contours of a face affect the activity in the IT area.
– Event-related components related to the superior temporal sulcus (STS) activity are larger when eyes are averted than when looking straight.
– The direction of the features of a face affects the face perception processing in the right hemisphere. On the other hand, the matching of the direction between the contours and features of a face affects the processing in the left hemisphere.
– Random dots blinking, which presents stimuli without a change in luminance during the presentation of a face, is a useful visual stimulus method to investigate face perception processing.

## Introduction

Facial information, such as sex, familiarity, and expression, plays an important part in our daily lives. By judging facial information, especially expressions, one can read and sympathize with others’ emotions through social interaction. In psychology, human face perception is one of the main topics. Therefore, many studies have been performed and models of human face perception were suggested (e.g., Bruce and Young, 1986).

In addition, there are many studies of human face perception using non-invasive methods for human subjects. Functional magnetic resonance imaging (fMRI) has an excellent spatial resolution that is sufficient to detect activated areas due to an increase in blood flow in the brain areas. Previous fMRI studies on human face perception revealed a specific area related to face perception, termed the fusiform face area (FFA) (for example, Kanwisher et al., 1997).

On the other hand, electroencephalography (EEG) has an excellent temporal resolution. In addition, high-density recordings can provide reasonably good spatial resolution. Therefore, EEG detects time sequences of human face perception. A well-known component related to human face perception is a negative component that occurs at approximately 170 msec after the presentation of a face, which is termed N170 (for example, Bentin et al., 1996). N170 is the most well-known component related to face perception because it is larger for faces than other objects (chair and car). Magnetoencephalography (MEG) also has the excellent temporal and spatial resolution and is equally useful to investigate the face perception process.

In this review, we have introduced a series of previous EEG and MEG studies on human face perception and discussed the following characteristics of face perception processing: (1) the inferior temporal (IT) activity evoked by a face (Watanabe et al., 1999a,b), (2) the effects of face contours and features on processing for face perception (Miki et al., 2007), (3) the effects of eye aversion on event-related potentials (ERPs) (Watanabe et al., 2002), (4) face inversion effects and differences between the right and left hemispheres in face perception (Watanabe et al., 2003; Miki et al., 2011), and (5) a useful visual stimulus method for investigating face perception (Miki et al., 2009).

## The Inferior Temporal Activity Evoked by a Face

A previous study using MEG (Watanabe et al., 1999a) investigated the temporal and spatial characteristic processes in human face perception. Twelve normal adult subjects with normal and corrected visual acuity participated in this study.

During MEG recording, 1M and 2M components were identified for face with opened eyes and face with closed eyes in both hemispheres. The 1M component was identified approximately 132 msec after the presentation of each stimulus and the activity in the occipital area related to primary visual perception represented by a change in luminance was estimated from the 1M component. The 2M component was identified approximately 179 msec after face stimulation (face with opened eyes and face with closed eyes) in ten subjects for each stimulus in the right but in only five subjects in the left hemisphere. In addition, there was no significant difference in the 2M latency between face with opened eyes and face with closed eyes. The activity in the IT area around the FFA related to face perception was estimated from the 2M component.

**FIGURE 1:**
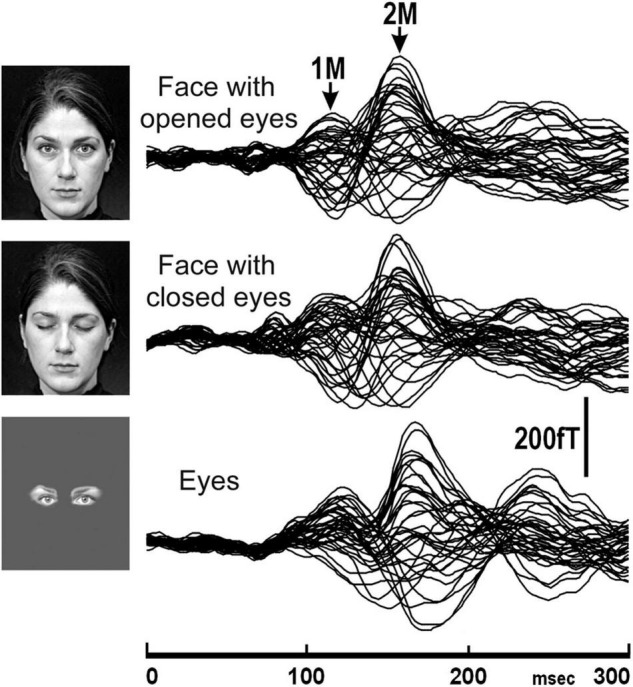
The MEG waveforms of a representative subject in response to the presentation of stimuli, face with opened eyes, face with closed eyes, and eyes recorded from the right hemisphere. The MEG waveforms recorded from 37 channels are superimposed [adopted from Watanabe et al. (1999b)]. MEG, magnetoencephalography.

Another previous MEG study (Watanabe et al., 1999b) investigated the difference in temporal and spatial characteristics of processes of perception of faces and eyes in humans. In this study, ten normal adult subjects with normal and corrected visual acuity had participated, and face with opened eyes, face with closed eyes, and eyes were presented (Figure 1).

In this study, 2M components were recorded for all subjects for each condition from the right hemisphere (Figure 1). On the other hand, a 2M component for eyes was recorded from the left hemisphere in all five subjects who had a clear 2M component for face with opened eyes and face with closed eyes. In both the right and left hemispheres, the 2M latency for eyes was significantly longer than for face with opened eyes and face with closed eyes.

The above studies (Watanabe et al., 1999a,b) suggested that the MEG components related to IT activity reflect the process of detection of a face.

## The Effects of Face Contours and Features on Processing for Face Perception

**FIGURE 2:**
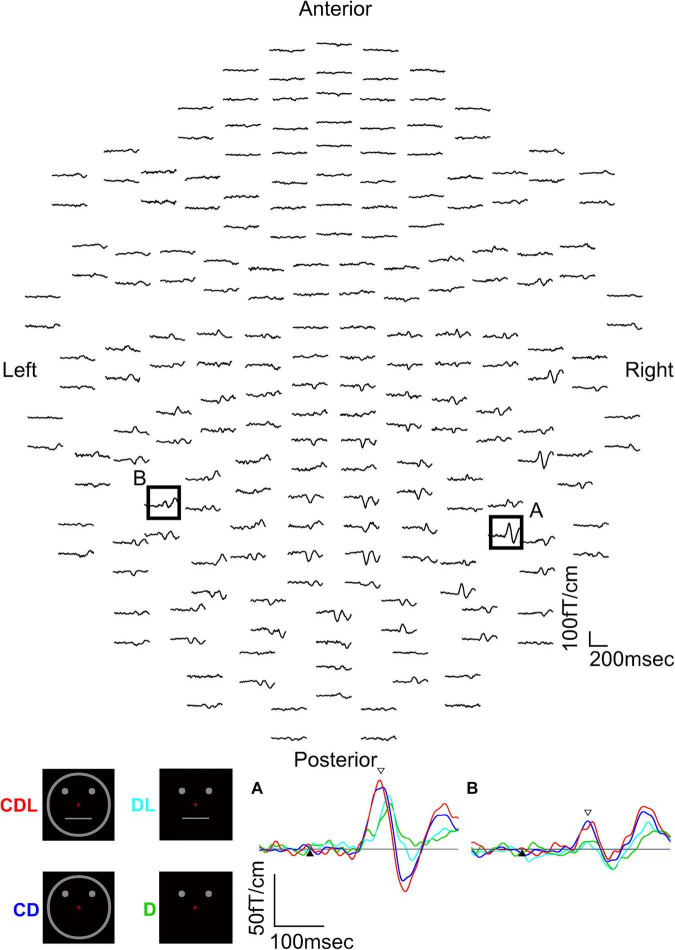
MEG waveforms (upper) of a representative subject following the stimulus onset in the CDL condition. MEG waveforms (lower) at sensors A and B in the upper image show a clear component recorded for CDL, CD, DL, and D in the right and left hemispheres. **(A)** Representative waveforms at sensor A in the right hemisphere of the upper image. **(B)** Representative waveforms at sensor B in the left hemisphere of the upper image. Black arrows show the stimulus onset and white arrows show the response selected for further analysis [adopted from Miki et al. (2007)]. MEG, magnetoencephalography; CDL, contour, dots, and line; CD, contour and dots; DL, dots and line; D, dots only.

The above studies (Watanabe et al., 1999a,b) revealed that the IT area is involved in the detection of a face and that a face is detected faster than eyes. However, it is unknown why a face is detected faster than eyes and whether facial contours and features other than eyes are necessary for the rapid detection of a face. Therefore, we used a schematic face and recorded MEG responses evoked in the IT area to investigate the effects of face contours and features on the detection of the face (Miki et al., 2007). We examined thirteen normal adult subjects with normal or corrected visual acuity and presented four visual conditions as follows (Figure 2): (1) contour, two dots, and a horizontal line (CDL): a schematic face having contour (circle), eyes (two dots), and a mouth (horizontal line) was presented, (2) contour and two dots (CD): the horizontal line was removed from CDL, (3) two dots and a horizontal line (DL): the circle was removed from CDL, and (4) two dots (D): only two dots were presented (Figure 2).

**FIGURE 3:**
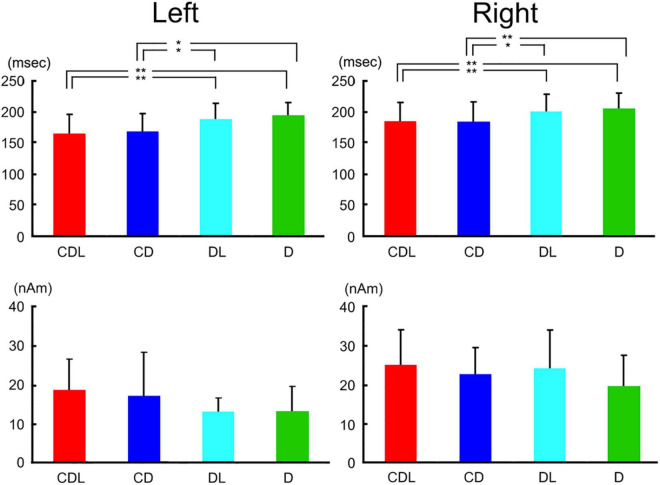
Bar graphs of peak latency (upper) and the maximum amplitude of dipole moment (lower) for all conditions after stimulus onset in the right and left hemispheres. Error bar shows the SD [adopted from Miki et al. (2007)]. *p < 0.05 and ^**^p < 0.01. SD, standard deviation.

The waveforms were recorded from 204 gradiometers by a 306-channel biomagnetometer in a representative subject following stimulus onset in CDL (Figure 2) and the waveforms in all conditions at representative sensors in each hemisphere of the same subject are shown in Figure 2. Using a single equivalent current dipole (ECD) model (Hämäläinen et al., 1993), ECDs were estimated in the IT area under all conditions for eleven of thirteen subjects in the right and left hemispheres. When the dipole moment of the estimated dipole was at a maximum after visual stimulus onset, the peak latency was significantly longer for DL and D than for CDL and CD in both hemispheres (Figure 3). On the other hand, regarding the maximum dipole moment of IT activity, it was larger for CDL than the others in both hemispheres, but there were no significant differences among conditions in either hemisphere due to the variability between individual subjects (Figure 3).

Previous ERP studies reported that it takes longer to recognize an image of the eyes only than the whole face (Bentin et al., 1996; Shibata et al., 2002; Itier et al., 2006). Moreover, Shibata et al. (2002) reported that the latency of N170 evoked by facial features (eye, nose, and mouth) was shorter with facial contours than without. Our study (Miki et al., 2007) is consistent with this study, suggesting that facial contour is one of the most important parts of face perception and shortens the perception process.

## The Effects of Eye Aversion on Event-Related Potentials

IT activity reflected the detection process of a face during face perception in our previous studies (Watanabe et al., 1999a,b), but we perceive facial information from facial parts (eyes and mouth). In particular, aversion of the eyes is highly important in social communication. The eye direction indicates attention to others or disgust, and we perceive others based on the direction of the eyes.

**FIGURE 4:**
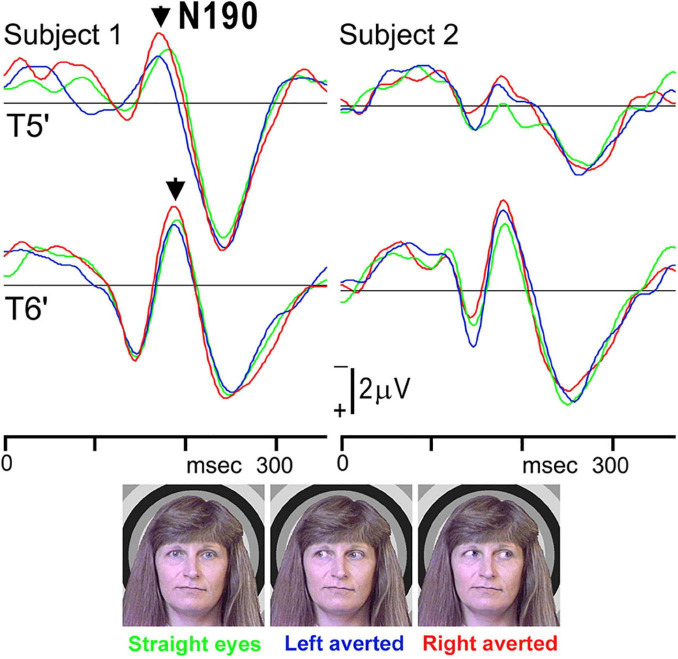
ERP waveforms in two representative subjects for straight eyes, left averted, and right averted conditions at T5′ and T6′ electrodes in the left and right temporal areas, respectively. A negative component, N190, was clearly identified for all stimuli [adopted from Watanabe et al. (2002)]. ERP, event-related potentials.

Therefore, we investigated whether eye direction indicates attention to others using faces with different eye directions in our previous study (Watanabe et al., 2002). Fourteen adult normal subjects with normal or corrected visual acuity had participated. Three visual stimuli were randomly presented (Figure 4): (1) straight eyes: a face with the eyes gazing at the viewer, (2) left averted: a face with the eyes averted to the left, and (3) right averted: a face with the eyes averted to the right. In ERPs for each condition, a large negative component (N190) at approximately 190–200 msec after stimulus onset was recorded at both the T5′ (left temporal area, 2 cm below T5 in the International 10–20 System) and T6′ (right temporal area, 2 cm below T6 in the International 10–20 System) electrodes (Figure 4). There were no significant differences among conditions in N190 latency, but N190 for right averted was significantly larger than that for straight eyes at the T6′ electrode (Table 1). N190 amplitudes were also larger for left averted at T6′ and for left and right averted at the T5′ electrode than for straight eyes, but the difference was not significant (Table 1). In addition, mean values for left averted and right averted were calculated as “averted eyes” and compared with those for straight eyes (Table 1). The peak latency did not significantly differ, but the amplitudes of N190 for averted eyes were larger than those for straight eyes at T5′ and significantly larger at T6′ electrodes (Table 1).

**TABLE 1:** Mean and SD of the maximum amplitude of the N190 component at T5′ and T6′ electrodes for straight eyes, left averted, right averted, and averted eyes (mean value of left averted and right averted).

	T5′	T6′
Straight eyes (μV)	−1.6 ± 0.8	−3.1 ± 2.4^a^
Left averted (μV)	−1.7 ± 0.9	−3.4 ± 2.7^a^
(%)	113.2 ± 52.4	113.4 ± 35.7
Right averted (μV)	−2.0 ± 0.9	−3.8 ± 2.8^a,b^
(%)	133.5 ± 61.2	126.4 ± 30.6^b^
Averted eyes (μV)	−1.8 ± 0.8	−3.6 ± 2.7^a,b^
(%)	119.3 ± 55.2	119.9 ± 24.6^b^

*In addition, values for the three averted conditions are expressed as percentages after normalization with straight eyes to clarify the effects of aversion in each hemisphere more precisely [adopted from Watanabe et al. (2002)].*

*^a^Inter-hemispheric difference: amplitude was significantly larger at the T6′ electrode than at the T5′ electrode (p < 0.05).*

*^b^Amplitude was significantly larger for right averted and averted eyes than straight eyes at the T6′ electrode (p < 0.01).*

*SD, standard deviation.*

The superior temporal sulcus (STS) plays a role in the perception of gaze direction, eye and mouth movement, and body movement according to previous studies (e.g., De Souza et al., 2005). An fMRI study revealed that the perception of eye direction is mediated more by the STS (Hoffman and Haxby, 2000) and that the STS is important in the perception of the changeable aspects in face information. On the other hand, in our previous studies (Watanabe et al., 1999a; Miki et al., 2004), the IT activity was not affected by information of eyes or mouth form (opened or closed) within a face. Thus, the larger ERPs evoked by eye aversion in the present study (Watanabe et al., 2002) may be related to STS activity rather than IT activity. In addition, the activity when gazing to the right was greater than when gazing to the left. The following possibility was hypothesized: the eyes averted to the right may have more of an effect in right-handed persons because all subjects in this study were right-handed.

## Face Inversion Effects and Differences Between the Right and Left Hemispheres in Face Perception

In previous psychophysical studies (e.g., Farah et al., 1995), people detected an inverted face slower and with more difficulty than an upright face. This phenomenon is termed the “face inversion effect” and it revealed that a process in face perception is specific to the upright direction.

**FIGURE 5:**
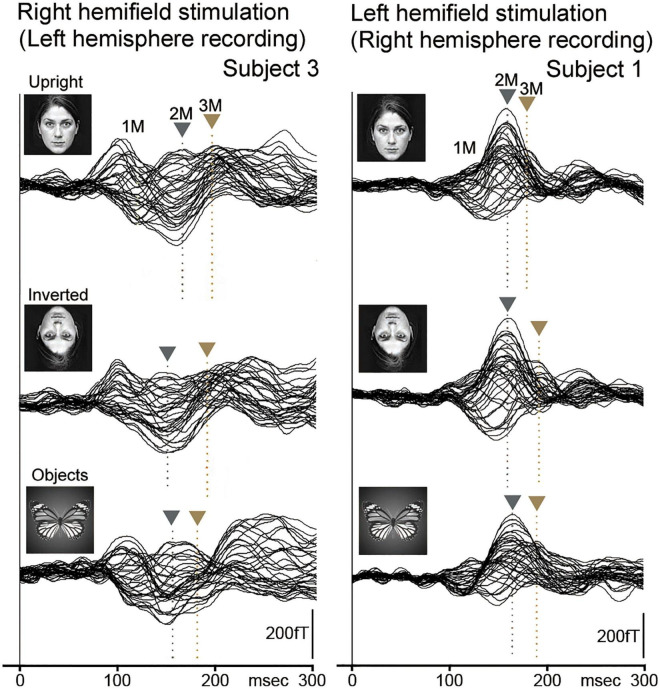
The MEG waveforms for LHF and RHF stimulation in two representative subjects. MEG components (1M, 2M, and 3M) were clearly recorded in the right (LHF stimulation) and left hemispheres (RHF stimulation) in upright (upright face), inverted (inverted face), and objects (butterfly) conditions. The vertical gray and brown arrows indicate the peaks of 2M and 3M, respectively. In addition, the vertical gray and brown dotted lines indicate the peak latencies of 2M and 3M, respectively. 2M and 3M latencies for objects were not significantly different from those for faces (upright and inverted) [adopted from Watanabe et al. (2003)]. MEG, magnetoencephalography; LHF, left hemifield; RHF, right hemifield.

Our MEG study (Watanabe et al., 2003) was performed to investigate evoked components by (1) upright faces (upright condition), (2) inverted faces (inverted condition), and (3) butterfly (objects condition) (Figure 5). Ten normal adult subjects with normal or corrected visual acuity had participated. For MEG recording, the sensor was placed covering temporo-occipital regions in the right and left hemispheres for left and right hemifield (LHF and RHF) stimulation, respectively, and right and left hemispheres were examined independently.

The evoked components (1M, 2M, and 3M) by each condition were identified, with the 2M component being the largest and most major among these components from the right and left hemispheres to LHF and RHF stimulations in representative subjects (Figure 5).

2M and 3M latencies were longer for the inverted condition than for the upright condition in the right hemisphere and shorter in the left hemisphere (Figure 5 and Table 2), although 2M and 3M latencies were not significantly different between upright and inverted conditions (Table 2). In addition, 2M and 3M latencies for the objects condition were not significantly different from those for upright and inverted conditions, and the 3M amplitude in the left hemisphere was significantly smaller for the objects condition than for upright and inverted conditions (Table 3).

**TABLE 2:** Mean and SD of peak latency (msec) of 2M and 3M components contralateral to the stimulated hemifield for upright (upright face), inverted (inverted face), and objects conditions [adopted from Watanabe et al. (2003)].

	Upright	Inverted	Objects
**Left hemifield stimulation (Right hemisphere)**			
2M	161.6 ± 11.8	164.3 ± 10.9	164.3 ± 11.3
3M	185.3 ± 15.4	187.3 ± 15.5	188.6 ± 13.7
**Right hemifield stimulation (Left hemisphere)**			
2M	155.1 ± 7.4	153.8 ± 5.8	157.0 ± 7.3
3M	183.5 ± 10.0	183.8 ± 9.0	183.9 ± 8.5

*SD, standard deviation.*

**TABLE 3:** Mean and SD of the maximum amplitude of 2M and 3M components contralateral to the stimulated hemifield.

	Upright	Inverted	Objects
**Left hemifield stimulation (Right hemisphere)**			
2M	134.0 ± 49.1 fT	101.3 ± 11.8%	98.5 ± 22.4%
3M	90.0 ± 53.1 fT	111.7 ± 31.1%	122.8 ± 43.2%
**Right hemifield stimulation (Left hemisphere)**			
2M	96.6 ± 40.7 fT	99.1 ± 9.9%	102.9 ± 25.1%
3M	88.8 ± 31.2 fT	89.6 ± 23.1%	65.5 ± 17.3%^a^

*For the upright (upright face) condition, MEG data are in fT. Values for inverted (inverted face) and objects conditions are expressed as percentages after normalization with maximum amplitudes for the upright condition [adopted from Watanabe et al. (2003)].*

*^a^The maximum amplitude for objects condition was significantly smaller than those for upright (upright face) (p < 0.001) and inverted (inverted face) (p < 0.01) conditions.*

*SD, standard deviation; MEG, magnetoencephalography.*

In multiple source modeling, the 3-dipole model (early visual area, IT area, and lateral temporal area) was the most appropriate for upright and inverted conditions, but not for the objects condition.

A previous study (McCarthy et al., 1999) revealed that upright faces are more rapidly processed in the right hemisphere, whereas inverted faces are more rapidly processed in the left, i.e., the right hemisphere had better processing of information about upright faces and the left had better processing of information about inverted faces. Consistent with the previous study (McCarthy et al., 1999), our study (Watanabe et al., 2003) revealed the following: (1) there was a difference in the face perception process between right and left hemispheres, (2) there were differences in processing between upright and inverted faces due to temporal processing differences, and (3) there were differences in brain areas related to processing between faces and objects.

There was a difference in the face perception process between the right and left hemispheres; however, it is unknown why there is such a difference and how upright and inverted faces were processed. We investigated the effects of inverting facial contours (hair and chin) and features (eyes, nose, and mouth) on processing for face perception using MEG (Miki et al., 2011).

**FIGURE 6:**
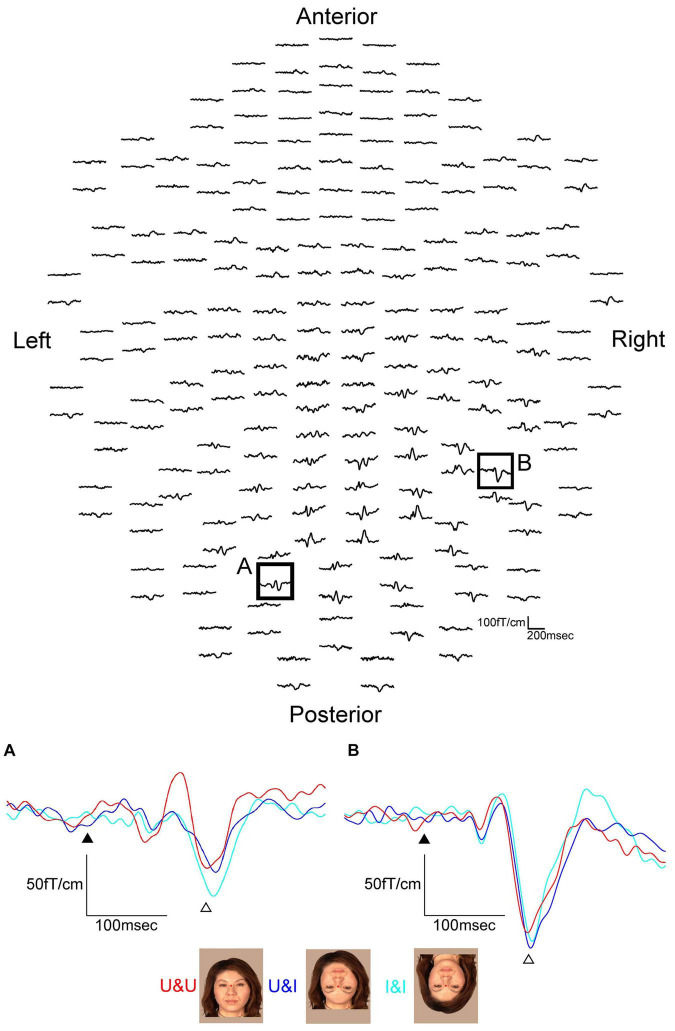
MEG waveforms (upper) of a representative subject following stimulus onset in the U&U condition. MEG waveforms (lower) at sensors A and B in the upper image show a clear component recorded for U&U, U&I, and I&I conditions in the left and right hemispheres. **(A)** Representative waveforms at sensor A in the left hemisphere of the upper image. **(B)** Representative waveforms at sensor B in the right hemisphere of the upper image. Black arrows show the stimulus onset and white arrows show the response selected for further analysis. Responses after the stimulus onset were longer in latency for U&I and I&I than for U&U in the right hemisphere, but longer in latency for U&I than for U&U and I&I in the left hemisphere in this subject [adopted from Miki et al. (2011)]. MEG, magnetoencephalography; U&U, upright contours and upright features; U&I, upright contours and inverted features; I&I, inverted contours and inverted features.

We examined ten normal adult subjects with normal or corrected visual acuity and presented three conditions as follows (Figure 6): (1) upright contours and upright features (U&U): contours (hair and chin) and features (eyes, nose, and mouth) upright, (2) upright contours and inverted features (U&I): contours remained upright, but the features were mirrored and inverted relative to the U&U condition, although the spatial relationship among the features was not changed, and (3) inverted contours and inverted features (I&I): A mirrored and inverted form of the image used in the U&U condition.

In both hemispheres, ECDs were located in the IT area, corresponding to FFA, for all conditions from all subjects. The waveforms were recorded from 204 gradiometers by a 306-channel biomagnetometer in a representative subject for the U&U condition and the waveforms in all conditions at representative sensors, where the largest component was identified for the U&U condition in each occipital or temporal area of the same subject, are shown in Figure 6.

**FIGURE 7:**
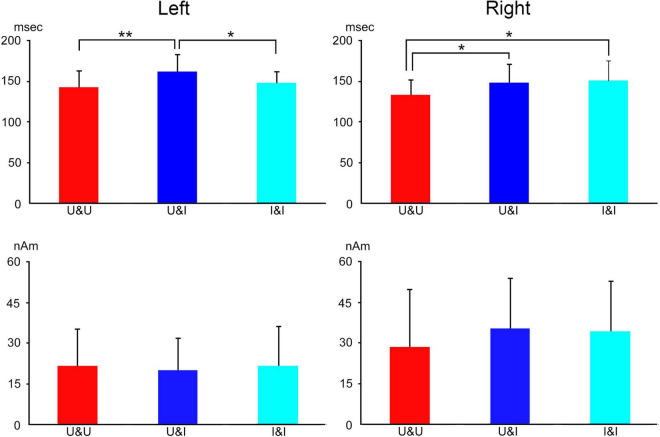
Bar graph of peak latency (upper) and the maximum amplitude of dipole moment (lower) for all conditions after stimulus onset in the right and left hemispheres. Error bar shows the SD [adopted from Miki et al. (2011)]. *p < 0.05 and ^**^p < 0.01. SD, standard deviation.

Concerning the peak latency of the ECD, the latency was significantly longer for U&I and I&I than for U&U in the right hemisphere and significantly longer for U&I than for U&U and I&I in the left hemisphere (Figure 7). On the other hand, regarding the maximum moment (strength) of the ECD, it was smaller for U&U than for U&I and I&I conditions in the right hemisphere, although there were no significant differences among stimulus conditions in either hemisphere due to individual differences (Figure 7).

In the U&U condition, the spatial relationship among the facial features (eyes, nose, and mouth) was intact and the features were upright; however, in the U&I and I&I conditions, the features were inverted but retained their spatial relationships even though the contours (hair and chin) of the face were upright or inverted. The U&U condition was considered “easier” to encode because it fits a normal template. In this study (Miki et al., 2011), the ECD was in both IT areas related to face perception, consistent with previous studies (e.g., Watanabe et al., 1999a,b). Based on previous studies (Watanabe et al., 1999a,b; Watanabe et al., 2003), we considered the right IT area related to face perception to be affected by the direction of features. On the other hand, in U&U and I&I conditions, the spatial relationships between the facial contours and features were intact whether the face was upright or inverted; however, in the U&I condition, they were disrupted. We considered the left IT area related to face perception to be affected by the disruption of the spatial relationships between the contours and features, different from the right hemisphere. This study (Miki et al., 2011) demonstrated the differences between the right and left hemispheres in face perception.

## A Useful Visual Stimulus Method for Investigating Face Perception

In previous studies that investigated face perception, the luminance differed after visual stimuli were presented and the occipital area related to the primary visual perception was activated by this change in luminance. Therefore, the IT area related to face perception cannot be completely separated from strong- and long-lasting activities in the primary visual areas.

A useful visual stimulus method, random dots blinking (RDB), uses temporal changes in patterns of many small dots to present stimuli without a change in luminance during the presentation of an object (e.g., a circle, a letter, or a schematic face), which reduces activities in the occipital area related to the primary visual perception, and detects only activity in the IT area related to visual form perception, such as faces and letters (Okusa et al., 1998; Okusa et al., 2000). In the RDB method, white dots are presented on a black background. Each dot turns white or black according to a pseudorandom plan and subjects perceive the white dots to be moving randomly. While presenting the stimuli, the dots forming stimuli remain unchanged, whereas other dots are changed, and subjects perceive the shape of the stimuli without a change in luminance.

**FIGURE 8:**
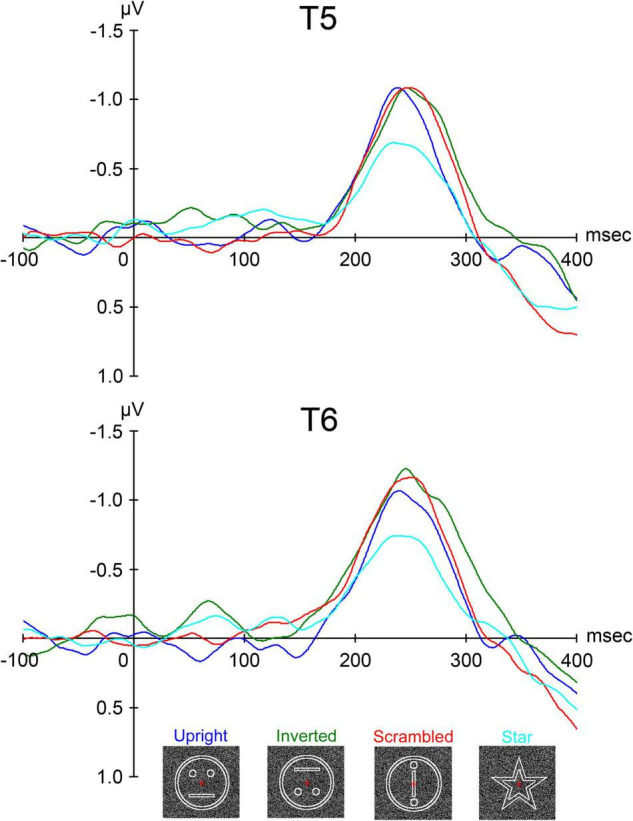
The grand-averaged waveforms from all ten subjects of ERP for upright (schematic face), inverted (the upright stimulus inverted), scrambled (the same contours and features as in upright but with the spatial relation distorted), and star (a star shape whose occurrence was enumerated and then reported after sessions by the subjects) conditions at the T5 and T6 electrodes in the left and right temporal areas, respectively [adopted from Miki et al. (2009)]. ERP, event-related potential.

We investigated the face perception process using the RDB method in ten normal adult subjects with normal or corrected visual acuity (Miki et al., 2009). We presented visual stimuli using RDB. Four stimuli were presented as follows (Figure 8): (1) upright: a schematic face consisting of a large circle, two dots, and a straight line. (2) inverted: the upright stimulus was inverted, but the spatial relationship between the contours and features was preserved. (3) scrambled: the spatial relationship between the contours and features was distorted. The scrambled stimulus was vertically and horizontally symmetrical because subjects were unable to judge upright or inverted. (4) star: subjects were asked to count the number of stars and to report it after the sessions in order to draw their attention to this stimulus and avoid paying attention to upright, inverted, and scrambled.

In a previous study using RDB (Okusa et al., 1998; Okusa et al., 2000), a clear evoked component peaking at around 200–300 msec was identified. In this study (Miki et al., 2009), we identified a component that had a peak deflection 200–300 msec after stimulus onset at T5 and T6 electrodes of the International 10–20 System in the left and right temporal areas, respectively. The peak latency when the amplitude of the component was maximal for each condition after the stimulus onset and the maximum amplitude of the component were individually measured within 300 msec.

The grand-averaged waveforms of evoked components after stimulus onset are shown in Figure 8. There was no component peaking at around 100 msec like P1 (or P100), which is considered to reflect the activities of the occipital areas related to the primary visual perception by luminance change in ERP studies. A clear negative component peaking at around 250 msec, termed N-ERP250, was identified at both the T5 (left) and T6 (right) electrodes in both temporal areas (Figure 8). At both electrodes, the latency of N-ERP250 was significantly shorter for upright than for inverted and scrambled and slightly shorter for upright than for star (Figure 8 and Table 4). In addition, the amplitude of N-ERP250 was significantly larger for upright, inverted, and scrambled than for star at the T6 electrode in the right hemisphere and larger for upright, inverted, and scrambled than for star at the T5 electrode, although there were no significant differences among conditions at the T5 electrode (Figure 8 and Table 4). We estimated the sources of N-ERP250 from the grand-averaged ERPs within 200–300 msec using brain electric source analysis as a multiple source model, and the estimated sources for each condition were located in the IT area, corresponding to FFA, related to face perception.

**TABLE 4:** The peak latency and maximum amplitude of N-ERP250 at T5 and T6 (the temporal area).

	T5 (Left)		T6 (Right)	
	(μV)	(msec)	(μV)	(msec)
Upright	−1.30 ± 1.09	246.7 ± 15.5	−1.43 ± 0.79^c^	247.8 ± 14.5
Inverted	−1.37 ± 1.18	258.3 ± 18.1^a^	−1.55 ± 0.95^d^	258.5 ± 18.7^b^
Scrambled	−1.43 ± 1.39	261.0 ± 17.5^a^	−1.58 ± 0.81^d^	259.4 ± 18.1^b^
Star	−1.03 ± 0.86	252.9 ± 20.7	−0.95 ± 0.64	251.2 ± 17.0

*Means and SD for upright, inverted, scrambled, and star [adopted from Miki et al. (2009)].*

*^a^p < 0.01 compared with upright.*

*^b^p < 0.05 compared with upright.*

*^c^p < 0.05 compared with star.*

*^d^p < 0.01 compared with star. SD, standard deviation.*

This study (Miki et al., 2009) revealed the following: (1) N-ERP250 is related to face perception, (2) the differences in N-ERP250 amplitude reflect differences between face perception and non-face perception processes, and (3) N-ERP250 is affected by not only the face direction but also the distortion in the spatial relation between the contours and features.

One previous study using intracranial ERP (Allison et al., 1999) reported that the first distinct response in visual ventral and lateral areas appears 200 msec after stimulus onset. In contrast, N-ERP250 in this study (Miki et al., 2009) was observed at a latency of 250 msec. Therefore, RDB stimuli activated a different subcortical pathway with slower conduction times before reaching the IT area.

## Discussion and Conclusion

In this review, we discussed the following characteristics of face perception processing based on our previous EEG and MEG studies.

First, event-related components related to activity in the IT area, corresponding to FFA, were evoked after the presentation of a face, and the detection process for a face was shorter than that for eyes because the face is important for social communication. The facial contours affect and shorten this process; therefore, they are one of the most important parts of face perception.

Second, event-related components related to the STS activity were larger when eyes were averted than when straight. This suggested that the direction of gaze, especially eye aversion, is an important indicator of an individual’s focus for automatic and unconscious attention.

Third, the face perception process was shortened by an upright face in the right hemisphere, whereas it was shortened by an inverted face in the left, revealing hemispheric differences in the face perception process. In addition, the direction of the features of a face affects the face perception processing in the right hemisphere, whereas matching of the direction between the contours and features of a face affects the processing in the left.

Lastly, using RDB without a change in luminance during the presentation of a face, the IT activity related to face perception was specifically detected without interference by activity in the occipital area related to the primary visual perception process. According to this method, the face perception process in the IT area is shorter for an upright face rather than for an inverted face and non-face stimuli.

## Author Contributions

KM and SW designed and performed the experiments, and performed data recording and statistical analyses in our previous studies. YT made the program used for the stimulus presentation in our previous studies. KM wrote this review. RK made suggestions about this review. KM had the overall responsibility for this review. All authors contributed to the article and approved the submitted version.

## Conflict of Interest

The authors declare that the research was conducted in the absence of any commercial or financial relationships that could be construed as a potential conflict of interest.

## Publisher’s Note

All claims expressed in this article are solely those of the authors and do not necessarily represent those of their affiliated organizations, or those of the publisher, the editors and the reviewers. Any product that may be evaluated in this article, or claim that may be made by its manufacturer, is not guaranteed or endorsed by the publisher.
